# Dental Mirror

**Published:** 1891-02

**Authors:** 


### BOOK NOTICES.

Dental Mirror. Rodriguez Ottolengui, Editor. Dental Publishing Company, 1300 Broadway, Room No. 16, New York City. Price, $1.00 per year.

This is a new journal devoted to dentistry. The sixth number is before us. It is full of articles highly interesting to the practical dentist. For example, we take "Comparative Methods," in which several eminent dentists, in answer to queries propounded by the editor, give their method of treatment for given defects of the teeth. W. M. J.

